# Health related quality of life and psychopathological distress in risk taking and self-harming adolescents with full-syndrome, subthreshold and without borderline personality disorder: rethinking the clinical cut-off?

**DOI:** 10.1186/s40479-017-0058-4

**Published:** 2017-05-07

**Authors:** Michael Kaess, Gloria Fischer-Waldschmidt, Franz Resch, Julian Koenig

**Affiliations:** 1 Section for Translational Psychobiology in Child and Adolescent Psychiatry, Department of Child and Adolescent Psychiatry, Centre for Psychosocial Medicine, University of Heidelberg, Blumenstraße 8, Heidelberg, 69115 Germany; 2 Department of Child and Adolescent Psychiatry, Centre for Psychosocial Medicine, University of Heidelberg, Heidelberg, Germany

## Abstract

**Background:**

Diagnostic standards do not acknowledge developmental specifics and differences in the clinical presentation of adolescents with borderline personality disorder (BPD). BPD is associated with severe impairments in health related quality of life (HRQoL) and increased psychopathological distress. Previously no study addressed differences in HRQoL and psychopathology in adolescents with subthreshold and full-syndrome BPD as well as adolescents at-risk for the development but no current BPD.

**Methods:**

Drawing on data from a consecutive sample of *N* = 264 adolescents (12–17 years) presenting with risk-taking and self-harming behavior at a specialized outpatient clinic, we investigated differences in HRQoL (KIDSCREEN-52) and psychopathological distress (SCL-90-R) comparing adolescents with no BPD (less than 3 criteria fulfilled), to those with subthreshold (3–4 BPD criteria) and full-syndrome BPD (5 or more BPD criteria). Group differences were analyzed using one-way analysis of variance with *Sidak* corrected contrasts or Chi-Square test for categorical variables.

**Results:**

Adolescents with subthreshold and full-syndrome BPD presented one year later at our clinic and were more likely female. Adolescents with subthreshold and full-syndrome BPD showed greater Axis-I and Axis-II comorbidity compared to adolescents with no BPD, and reported greater risk-taking behaviour, self-injury and suicidality. Compared to those without BPD, adolescents with subthreshold and full-syndrome BPD reported significantly reduced HRQoL. Adolescents with sub-threshold BPD and those with full-syndrome BPD did not differ on any HRQoL dimension, with the exception of *Self-Perception*. Similar, groups with sub-threshold and full-syndrome BPD showed no significant differences on any dimension of self-reported psychopathological distress, with the exception of *Hostility*.

**Conclusions:**

Findings highlight that subthreshold BPD in adolescents is associated with impairments in HRQoL and psychopathological distress comparable to full-syndrome BPD. Findings raise awareness on the importance of early detection and question the diagnostic validity and clinical utility of existing cut-offs. Findings support a lower diagnostic cut-off for adolescent BPD, to identify those at-risk at an early stage.

## Background

Borderline Personality Disorder (BPD) affects about 1–2% [1, 2] of the general population and is the most common personality disorder in clinical settings [3]. BPD is characterized by pathological personality traits in the domains of negative affectivity, emotional liability, anxiousness, separation insecurity or depressivity and behavioral characteristics such as disinhibition (i.e., impulsivity and risk taking) and antagonism (hostility) [3, 4]. BPD is a severe mental disorder, associated with functional impairment, a high suicide rate, other psychiatric comorbidities and personality disorders, extensive use of mental health services, high social and economic costs, and burden on families and care providers [4]. Diagnosing BPD in youth under the age of 18 has been discussed controversially [5] for different reasons [6]. However, the legitimacy of the BPD diagnosis in adolescents is nowadays widely acknowledged [7–12], as reflected in treatment guidelines and diagnostic manuals, including the Diagnostic and Statistical Manual for Mental Disorders, Fifth Edition (DSM-5) and the revision of International Classification of Disease 11^th^ edition (ICD-11) [3, 13]. Full-syndrome BPD is defined in the case that an individual meets five of the nine criteria proposed in the DSM-5 [3].

Major diagnostic classification systems have not yet adopted youth adequate criteria of BPD, focusing on developmental characteristics of BPD and differences in the clinical presentation of adolescents [6]. Adolescent BPD is frequently characterized by an over representation of acute symptoms [11, 14] – in particular risk-taking and self-harming behavior, that present important developmental trajectories for BPD [15] – and two of the nine DSM-5 diagnostic criteria. Self-injury (i.e., the intentional, self-directed act of injuring one’s own body tissue), itself is a common phenomenon among other risk-behaviors in adolescents [16, 17].

Studies addressing the validity of the DSM-5 diagnostic cut-off for BPD in adolescents are rare. Studies in population based and inpatient samples of adolescents suggest a single continuous dimension underlying BPD, that accounts for co-variation among diagnostic criteria [18, 19]. While the dimensional assessment of personality disorder severity has several advantages above categorical approaches, clinical decision making frequently relies on distinct clinical cut-offs. A compromise has been suggested by Zimmermann et al. [20], who argued, that a 3-point dimensional convention (absent, subthreshold traits, present) is as valid as more fine-grained approaches and has advantages compared to dichotomous diagnosis. Zimmermann et al. [20] suggested to score patients with personality disorders as subthreshold if they reported at least one trait of the disorder. Such approach seems particular fruitful for the clinical identification of adolescents at-risk for the development of BPD.

Clinical cut-offs need to be tested against external validators to proof validity and clinical utility. Asides measures addressing general psychopathological distress and comorbidity, dimensions of an individual patients’ health related quality of life (HRQoL) gain increasing attention in psychiatric research, providing a patient perspective on the severity of pathology and effectiveness of interventions [21–23]. In BPD, adults show significant impairments in HRQoL [24–26], in particular when comorbid with posttraumatic stress disorder (PTSD) [27], and studies have shown that BPD itself (independent of Axis-I comorbidity) predicts substantial impairment in HRQoL [28].

Here we aimed to adopt a 3-point dimensional approach for the diagnosis of BPD in adolescents, addressing differences in psychiatric comorbidity, risk-taking behavior, subjective psychopathological distress and HRQoL comparing adolescents with risk-taking and self-harm behavior and/or self-injury at-risk for the development of BPD to those with some BPD symptoms (subthreshold) and full-syndrome BPD. Given the over representation of risk-taking and self-harming behavior in adolescence with BPD, we adopted a diagnostic threshold of at least 3 traits for subthreshold BPD and utilized the regular DSM-5 convention [3] for the diagnosis of full-syndrome BPD.

In summary, the present study aimed to investigate differences in the clinical presentation of adolescents with risk-taking and self-harming behavior with full-syndrome, subthreshold and without BPD to clarify the validity of the existing DSM-5 diagnostic cut-off in youth BPD and to gain better insights into subjective domains of functioning and psychopathological impairment associated with subthreshold traits of the disorder in this age group. Based on clinical experience, it was hypothesized that adolescents with subthreshold BPD show greater psychopathological distress and diminished HRQoL compared to adolescents with no BPD, and that psychopathological distress and HRQoL would further differ between adolescents with sub-threshold and full-syndrome BPD. It was hypothesized that adolescents with full-threshold BPD show greater psychopathological distress and diminished HRQoL compared to adolescents with sub-threshold BPD.

## Methods

### General procedures

Data for the present analysis were collected in a consecutive help-seeking cohort of adolescents presenting at the specialized outpatient clinic for risk-taking and self-harm behavior (AtRiSk; *Ambulanz für Risikoverhalten & Selbstschädigung*) at the Clinic for Child and Adolescent Psychiatry, Centre of Psychosocial Medicine, University of Heidelberg. The ATR!Sk cohort study was approved by the Ethical Committee of the Medical Faculty, Heidelberg University, Germany (Study: ID S-449/2013) and carried out in accordance with the declaration of Helsinki [29]. All patients and their legal guardians provided written informed consent. In AtR!Sk, youth between 12 and 17 years of age with diverse risk-taking and self-harming behavior are clinically assessed and referred to subsequent treatment. To be included in the scientific evaluation of the outpatient clinic, adolescents have to report any recent engagement in risk-taking (i.e., binge-drinking, substance abuse, excessive media or Internet use, sexual risk behavior, delinquent behavior) or self-harm (non-suicidal self-injury or suicide attempts). Since June 2013 a total of 340 adolescents presented at AtR!Sk. From this consecutive baseline sample (first presentation at AtR!Sk) 303 (89.1%) were included in the scientific evaluation, according to the inclusion criteria, and provided written informed consent. At the time of analysis (September 2016), data were available for N = 266 (87.8%). Of these, only those with a complete assessment of BPD were included in the present analysis (n = 264, 99.2%). Two patients were excluded because their BPD diagnostic assessment was missing or incomplete. All data were collected within routine clinical care without any a-priori formulated research question.

### Clinical assessments

Psychiatric diagnoses were obtained using the German version of the *Mini-International Neuropsychiatric Interview for Children and Adolescents* (M.I.N.I-KID 6.0) [30, 31]. The M.I.N.I.-KID is a short structured diagnostic interview for DSM-IV and ICD-10 psychiatric disorders for children and adolescents aged 6–19 years. In addition, the German version of the *Structured Clinical Interview for DSM-IV-Axis II* (SCID-II) was used to assess borderline, avoidant, dependent and antisocial personality disorder [32]. Albeit the SCID-II has been validated in adults [32], it is suitable for the use in adolescents [10, 33]. *The German version of the Self-Injurious Thoughts and Behavior Interview* (SITBI-G, [34]) was used for the detailed assessment of NSSI and suicide attempts [35]. The SITBI-G is a semi-structured interview for the assessment of self-injurious thoughts and behaviors, and shows excellent psychometric properties. To meet DSM-5 criteria for NSSI, the SITBI was slightly modified assessing the days of engagement in NSSI. The SITBI has been validated in adolescents (12–19 years) [34]. All interviews were carried out by a team of trained and experienced clinicians. Inter-rater reliability (IRR) of the diagnostic interviews based on audio recordings taken from a sub-set of diagnostic interviews is assessed on a yearly basis. Based on the latest IRR assessment in November 2016, conducted on *n* = 47 audio recordings rated by two independent raters, the individual interclass correlation coefficient (ICC) for the evaluation of the number of BPD criteria met (critical for the present analysis) was ICC = .935 (95%CI: .887; .963). On the individual criterion level, agreements between raters ranged from 87.23% (criterion 2, ϰ = .744; SE = .146) to 97.87% (criterion 7, ϰ = .953; SE = .146). Diagnostic agreement (full-threshold BPD) was at 93.62% (ϰ = .872; SE = .146). Single items of the *Life Problems Inventory* [36], a measure to quantify borderline personality features in adolescents, were used to quantify other risk behavior, including se*x with people barely knew*, *drinking too much alcohol*, *drug consumption*, *delinquent behavior/breaking the law*. Each item is rated on a 5-point Likert-type scale with the anchor points: 1 – *not at all like me*, 2 – *a little bit like m*e, 3 – *somewhat like me*, 4 – *quite a bit like me*, and 5 – *extremely like me*.

### Health related quality of life

The German 52-item self-report version of the KIDSCREEN generic HRQoL measure for children and adolescents (8–18 years of age) was used [37]. It measures 10 related quality of life dimensions, including: *Physical*- (5 items), *Psychological Well-Being* (6 items), *Moods and Emotions* (7 items), *Self-Perception* (5 items), *Autonomy* (5 items), *Parent Relations and Home Life* (6 items), *Social Support and Peers* (6 items), *School Environment* (6 items), *Social Acceptance (Bullying)* (3 items), and *Financial Resources* (3 items). Most items are scored on 5-point Likert-type scale. T-values based on Rasch person parameter are calculated for each dimension.

### Psychopathological distress

The German version [38] of the Symptom Checklist-90-R (SCL-90-R) [39, 40] was used as self-report measure of psychopathological distress. The SCL-90-R was developed and validated for its use in participants 13 years and older. Cronbach’s Alpha was assessed for each scale used for the present analysis. The 90 items of the SCL-90-R cover 9 principal symptom dimensions including: *Somatization* (12 items; α = .887), *Obsessive-Compulsive* (10 items; α = .838), *Interpersonal Sensitivity* (9 items; α = .869), *Depression* (13 items; α = .910), *Anxiety* (10 items; α = .876), *Hostility* (6 items; α = .778), *Phobic Anxiety* (7 items; α = .830), *Paranoid Ideation* (6 items; α = .782), and *Psychoticism* (10 items; α = .825). A *Global Severity Index* (GSI; α = .976) can be derived. Each item is scored on a 0 to 4 Likert-type scale. Symptom dimensions and the GSI are derived by the mean across included items (values ranging from 0 to 4).

### Statistical analysis

Groups were formed based on the structured BPD assessment, distinguishing adolescents with risk-taking and/or self-injury and no BPD (less than 3 BPD criteria), sub-threshold (3 or 4 BPD criteria) and full-syndrome BPD (5 or more BPD criteria fulfilled). Group differences on all dependent variables were analyzed using one-way analysis of variance with *Sidak* corrected contrasts for continuous variables and Chi-Square test for dichotomous or categorical data. Ordered logistic regression was used to compute Sidak corrected post-hoc comparisons from significant Chi-Square tests. Mixed linear-regression was used in subsequent analysis addressing group differences on the two main outcomes (HRQoL and psychopathological distress) to adjust for group difference on sex and age. All analyses were performed using Stata/SE (Version 14.0; StataCorp LP, College Station, TX, US) with α set to .05. Graphs were prepared using GraphPad Prism (version 6.0, GraphPad Software Inc., USA).

## Results

### Sociodemographic characteristics

Sociodemographic characteristcs of the treatment seeking consecutive sample are provided in Table 1. Groups based on BPD criteria differed on sex (*χ* ^*2*^ = 34.670, *p* < *.0001*). Post-hoc tests showed significant differences between adolescents with full-syndrome BPD and subthreshold BPD (z = −1.91, *p = .003*), full-syndrome BPD and no BPD (z = −4.77, *p < .0001*), but not subthreshold BPD and adolescents with no BPD (z = −0.79, *p = .093*). Groups further differed on age (F_(2;261)_ = 6.67, *p = .002*). Pairwise comparisons showed that patients with full-syndrome BPD were significantly older than those with no BPD (MD: 0.78, *p = .001*). Patients with subthreshold and full-syndrome BPD (MD: 0.36, *p = .214*), as well as patients with no BPD and those with subthreshold BPD (MD: 0.42, *p = .192*) did not differ on age. Compared to patients with no BPD, patients with full-syndrome BPD were less likely to live with their biological mother (z = −2.44, *p = .043*). There were no significant differences between adolescents with subthreshold BPD and full-syndrome BPD (z = −0.16, *p = .998*), and subthreshold BPD and no BPD (z = −2.25, *p = .072*). Groups did not differ on any of the other sociodemographic variables.

**Table 1:** Sociodemographic Characteristcs of the Study Sample

Variable	no BPD	sub BPD	BPD	*p*
n (%)	72 (27.27)	83 (31.44)	109 (41.29)	
female, *n (%)*	46 (63.89)	66 (79.52)	105 (96.33)	*<.0001*
age, *years*	14.60 (1.51)	15.01 (1.49)	15.38 (1.28)	*.002*
First psychiatric contact, *years since*	1.02 (2.14)	1.19 (2.57)	1.97 (3.27)	*.098*
First psychiatric presentation, n (%)	37 (66.07)	41 (66.13)	40 (51.28)	*.116*
School, *n (%)*				*.929*
*Hauptschule*	9 (12.68)	9 (10.84)	10 (9.17)	
*Realschule*	22 (30.99)	30 (36.14)	41 (37.61)	
*Gymnasium*	28 (39.44)	27 (32.53)	37 (33.94)	
*other*	12 (16.90)	17 (20.48)	21 (19.27)	
relationship parents, *n (%)*				*.968*
*living together*, *n (%)*	29 (40.28)	35 (42.17)	43 (39.45)	
*separated/divorced*	35 (4.,61)	44 (53.01)	58 (53.21)	
*death of one parent*	3 (4.17)	2 (2.41)	4 (3.67)	
*never lived together*	2 (2.78)	1 (1.20)	3 (2.75)	
*unknown*	2 (2.78)	1 (1.20)	1 (0.92)	
living with biological mother, *n (%)*	62 (89.86)	61 (75.31)	75 (74.26)	*.021*
living with biological father, *n (%)*	30 (54.55)	37 (52.11)	44 (50.00)	*.868*

*values* represent means and standard deviations (SD) in brackets, unless otherwise indicated; p values refer to one-way ANOVA (continuous data) or Chi-Square tests for categorical/dichotomy data; *First psychiatric contact: time in years since first presentation at any given professional psychiatric facility (outpatient, inpatient); First psychiatric presentation: n of patients with first presentation at a psychiatric facility within our specialized outpatient clinic (*i.e.*, first contact same year of diagnostic interview); Type of School: After four years of elementary school the German school system branches into three types of secondary schools. The so called “Hauptschule” (Secondary General School which takes five years after Primary School) prepares pupils for vocational training, whereas the “Realschule” (Intermediate Secondary School) concludes with a general certificate of secondary education after six years. Eight years of “Gymnasium” provide pupils with a general university entrance qualification. Some missing data on relationship status of parents, living situation, as indicated by percentage values; some missing data on history of psychiatric presentation, as indicated by percentage values*

### Clinical characteristics

**Fig. 1:**
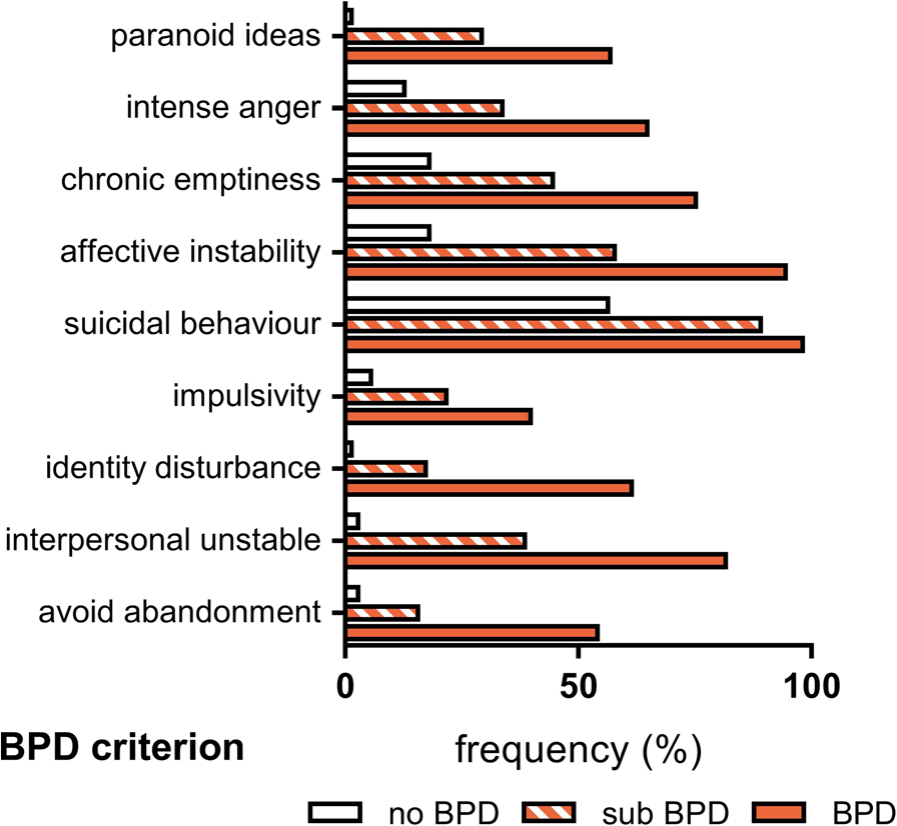
Relative Frequency of Single Fulfilled BPD Criteria by Group; BPD criteria according to DSM-5 [3]; frequency in percent based on total n by group

Patients with no BPD fulfilled on average 1.18 ± 0.8 BPD criteria. Adolescents with subthreshold and full-syndrome BPD fulfilled 3.5 ± 0.5 and 6.2 ± 1.3 respectively. The relative frequency of particular BPD criteria fulfilled is illustrated in Fig. 1.

**Fig. 2:**
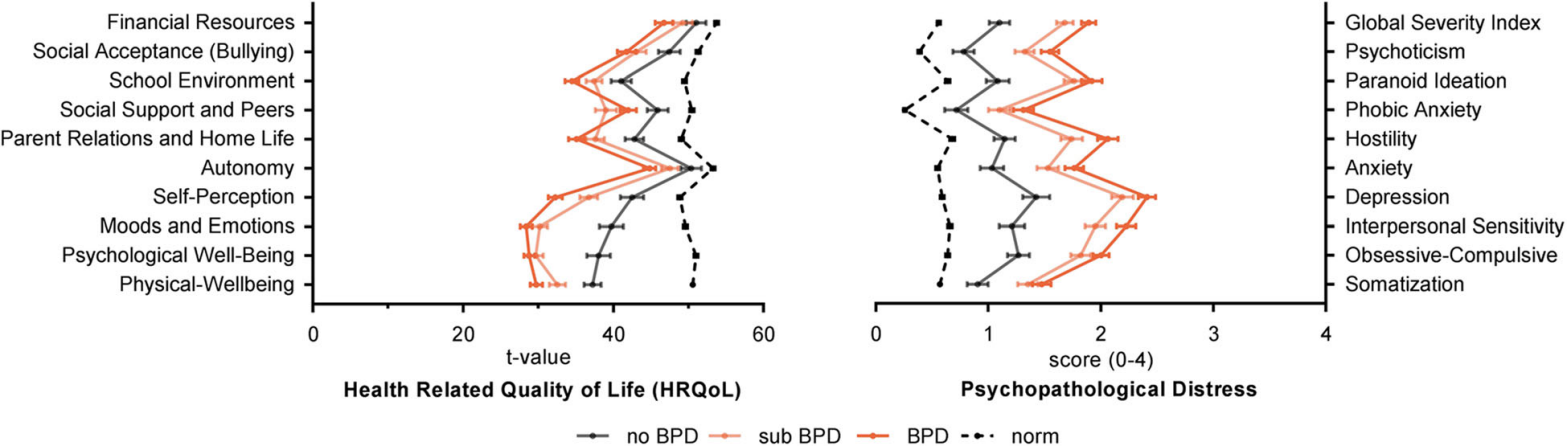
Health Related Quality of Life and Psychopathological Distress by Group; norm refers to norm HRQoL data from a German sample of male and female adolescents (n = 1091 to 1102) aged 12–17 years from the European KIDSCREEN-52 sample as provided in Appendix A7_B on Page 62 of the KIDSCREEN manual [56]; as well as SCL-90-R norm data from a German sample of male and female adolescents (n = 857) aged 12–17 years from the *Bremer Jugendstudie* [57] as provided in the German manual of the SCL-90-R on page 206 [58]; norm means are given for illustrative purposes only

Comorbid diagnoses according to ICD-10 (excluding F6X and F0X) were frequent (Fig. 2). Mood disorders (F30-39) were most frequent (n = 184) followed by neurotic, stress-related and somatoform disorders (F40-48, n = 128), followed by mental and behavioral disorders due to psychoactive substance use (F10-19, n = 73) and behavioral syndromes associated with physiological disturbances and physical factors (F50-59, n = 44). Groups significantly differed with respect to the average number of comorbid diagnosis (F_(2;261)_ = 14.06, *p* < .0001). On average, patients with full-syndrome BPD fulfilled diagnostic criteria for 2.17 (SD = 1.49) comorbid diagnoses followed by patients with subthreshold BPD with 1.99 (SD = 0.99) comorbid diagnoses, followed by patients without BPD with an average of 1.24 (SD = 0.81) comorbid diagnoses. Pairwise comparisons were significant for adolescents with subthreshold (MD = 0.75, *p* < .0001) and full-syndrome BPD (MD = 0.93, *p* < .0001) compared to those with no BPD, but not when comparing adolescents with subthreshold and full-syndrome BPD (MD = 0.18, *p* = .666).

With respect to the other personality disorders, groups differed on the presence of comorbid avoidant personality disorder (*χ* ^*2*^_*(262)*_ = 8.550, *p* = *.014*), that was more frequent in adolescents with full-syndrome BPD (26.17%) compared to adolescents with no BPD (9.72%; z = 1.19, *p = .026*). Other pairwise comparisons showed no significant differences. 15.66% of patients with subthreshold BPD fulfilled diagnostic criteria for avoidant personality disorder. Groups showed no differences with respect to the presence of dependent personality disorder (*χ* ^*2*^_*(246)*_ = 1.980, *p* = *.372;* 1.38 to 8.5%), but antisocial personality disorder (*χ* ^*2*^_*(261)*_ = 6.350, *p* = *.042*), that was more frequent in adolescents with full-syndrome BPD (9.43%), compared to those with no BPD (1.39%) and adolescents with subthreshold BPD (2.41%). However, pairwise comparisons showed no significant differences. Adolescents with subthreshold and full-syndrome BPD were more likely to engage in self-injury (Table 2). Groups significantly differed on the reporting of self-injury (*χ* ^*2*^_*(264)*_ = 43.539, *p* < .0001). Adolescents with subthreshold (94.0%) and full-syndrome BPD (100%) were more likely to report lifetime self-injury, compared to those with no BPD (70.8%). Differences between adolescents with subthreshold and no BPD were statistically significant (z = 3.51, *p = .001*) [100% in BPD prohibited adequate post-hoc comparison]. Groups significantly differed on acts of self-injury among those reporting lifetime self-injury during the past 12 months (F_(2;232)_ = 3.80, *p = .024*). Adolescents with full-syndrome BPD reported more acts of self-injury compared to adolescents with subthreshold BPD (MD: 37.06, *p = .032*). Patients with no BPD did not differ from adolescents with subthreshold (MD: −6.33, *p = .978*) or full-syndrome BPD (MD: 30.73, *p = .185*). Groups showed no significant differences with respect to the onset of self-injury (F_(2;235)_ = 0.24, *p = .790*). Groups significantly differed on the reporting of lifetime suicide attempts (*χ* ^*2*^_*(263)*_ = 42.170, *p* < .0001), with 19.72% of those with no BPD, 51.8% of the sub-threshold BPD group, and 67.89% of the full-syndrome BPD group reporting at least one lifetime suicide attempt. Differences were significant between adolescents with no BPD and those with subthreshold (z = 3.86, *p < .0001*) and full-syndrome (z = 5.95, *p < .0001*) BPD respectively. Further, adolescents with subthreshold and full-syndrome BPD showed significant differences in lifetime suicide attempts (z = 2.41, *p = .047*). The number of suicide attempts in those reporting prior attempts did not differ between groups (F_(2;128)_ = 0.16, *p = .849*). Groups showed significant differences on risk-taking behavior, including engaging in sex with people they barely knew (*χ* ^*2*^_*(243)*_ = 12.02, *p* = .002), drinking too much alcohol (*χ* ^*2*^_*(247)*_ = 19.91, *p* < .0001), drug consumption (*χ* ^*2*^_*(244)*_ = 18.82, *p* < .0001), and delinquent behavior (*χ* ^*2*^_*(247)*_ =6.84, *p* = .033). Only adolescents with full-syndrome BPD reported significant differences with respect to the engagement in sex with people they barely knew compared to adolescents with no BPD (z = 3.13, *p = .005*). Adolescents with subthreshold BPD did not differ from those with full-syndrome (z = 1.99, *p = .134*) or no BPD (z = 1.36, *p = .436*). Compared to those with no BPD (z = 4.13, *p < .0001*) and subthreshold BPD (z = 2.59, *p = .028*), adolescents with full-syndrome BPD were more likely to report drinking too much alcohol. Differences between adolescents with no BPD and subthreshold BPD were not significant (z = 1.75, *p = .223*). With respect to drug abuse, those with full-syndrome BPD (z = 3.85, *p < .0001*) significantly differed to adolescents with no BPD. Adolescents with subthreshold BPD did not differ from controls (z = 2.33, *p = .059*). Differences between adolescents with subthreshold and full-syndrome BPD were not significant (z = 1.89, *p = .167*). Similar, only adolescents with full-syndrome BPD reported greater delinquent behavior compared to adolescents with no BPD (z = 2.55, *p = .032*). Adolescents with subthreshold BPD did not differ from those with full-syndrome (z = 1.21, *p = .540*) or no BPD (z = 1.40, *p = .413*).

**Table 2:** Risk-Taking Behavior by Group

Risk Behavior	no BPD	sub BPD	BPD	*p*
Self-injury				
*lifetime, yes n (%)*	51 (70.83)	78 (93.98)	109 (100.00)	*< .0001*
*past 12 months, days*	67.55 (137.53)	61.22 (69.48)	98.28 (91.13)	*.024*
age of onset*, years*	12.80 (1.51)	13.09 (2.01)	12.94 (1.75)	*.790*
*Suicide attempts*				
*lifetime, yes n (%)*	14 (19.72)	42 (50.60)	74 (67.89)	*< .0001*
*lifetime attempts*	4.86 (5.99)	7.83 (24.62)	6.51 (14.18)	*.849*
*Sex with people barely knew, n (%)*				*.002*
*not at all/ a little bit like me*	58 (89.23)	64 (84.21)	78 (76.47)	
*somewhat like me*	7 (10.77)	6 (7.89)	8 (7.84)	
*quite a bit/ extremely like me*	0 (0.00)	6 (7.89)	16 (15.69)	
*Drinking too much, n (%)*				*< .0001*
*not at all/ a little bit like me*	64 (95.52)	63 (82.89)	68 (65.38)	
*somewhat like me*	2 (2.99)	5 (6.58)	14 (13.46)	
*quite a bit/ extremely like me*	1 (1.49)	8 (10.53)	22 (21.15)	
*Drug consumption, n (%)*				*< .0001*
*not at all/ a little bit like me*	62 (93.94)	63 (84.00)	78 (75.73)	
*somewhat like me*	2 (3.03)	6 (8.00)	5 (4.85)	
*quite a bit/ extremely like me*	2 (3.03)	6 (8.00)	20 (19.42)	
*Delinquent behavior / breaking the law, n (%)*				*.033*
*not at all/ a little bit like me*	55 (83.33)	61 (79.22)	71 (68.27)	
*somewhat like me*	2 (3.03)	4 (5.19)	16 (15.38)	
*quite a bit/ extremely like me*	9 (13.64)	12 (15.58)	17 (16.35)	

*values* represent means and standard deviations (SD) in brackets, unless otherwise indicated; *p* values refer to one-way ANOVA (continuous data) or Chi-Square tests for categorical/dichotomy data; *Some missing data on risk-behaviour items, as indicated by percentage values; item response have been collapsed for readability (not at all like me and a little bit like me; quite a bit like me and extremely like me), analyses were performed on full 5-point scales*

### Health related quality of life

Groups significantly differed on all HRQoL dimensions, except for *Financial Resources* (F_(2;252)_ = 2.29, *p = .056*), including *Physical-Well-Being* (F_(2;252)_ = 14.13, *p < .0001*), *Psychological Well-Being* (F_(2;253)_ = 21.28, *p < .0001*), *Moods and Emotions* (F_(2;256)_ = 28.02, *p < .0001*), *Self-Perception* (F_(2;256)_ = 18.25, *p < .0001*), *Autonomy* (F_(2;255)_ = 6.71, *p = .001*), *Parent Relations and Home Life* (F_(2;248)_ = 10.78, *p < .0001*), *Social Support and Peers* (F_(2;254)_ = 6.44, *p = .002*), *School Environment* (F_(2;199)_ = 8.80, *p < .0001*), and *Social Acceptance/Bullying* (F_(2;252)_ = 4.72, *p = .010*). Group differences are illustrated in *Figure 3* and descriptive statistics including pairwise contrasts and the respective effect size estimates are provided in Table 3. In mixed-linear regression analysis, all main effects of group remained after adjusting for sex and age with the exception of the HRQoL domain of *Autonomy* that did not differ between adolescents with sub-threshold and no BPD. Sex – but not age - had a significant effect on all HRQoL domains of *Physical-Well-Being, Psychological Well-Being, Self-Perception,* and *Autonomy.* Male adolescents reported greater HRQoL in the respective domains.

**Table 3:** Group Contrast on Health Related Quality of Life (HRQoL); Sidak corrected contrasts from one way analysis of variance

Domain of HRQoL	no BPD vs. subthreshold BPD			no BPD vs. full-syndrome BPD			subthreshold BPD vs. full-syndrome BPD		
	MD	*ES*	*p*	MD	*ES*	*p*	MD	*ES*	*p*
*Physical-Wellbeing*	−4.69	*.49*	*.005*	−7.49	*.85*	*< .0001*	−2.79	*.31*	*.114*
*Psychological Well-Being*	−8.39	*.75*	*< .0001*	−9.24	*.92*	*< .0001*	−0.84	*.10*	*.914*
*Moods and Emotions*	−9.52	*.83*	*<.0001*	−11.32	*1.06*	*< .0001*	−1.80	*.21*	*.545*
*Self-Perception*	−5.77	*.48*	*.004*	−10.21	*.92*	*< .0001*	−4.45	*.44*	*.019*
*Autonomy*	−2.83	*.26*	*.212*	−5.53	*.57*	*.001*	−2.70	*.30*	*.180*
*Parent Relations and Home Life*	−5.21	*.49*	*.009*	−7.70	*.74*	*.000*	−2.49	*.23*	*.310*
*Social Support and Peers*	−6.88	*.56*	*.001*	−3.91	*.34*	*.097*	2.97	*-.25*	*.250*
*School Environment*	−3.62	*.35*	*.078*	−6.56	*.66*	*< .0001*	−2.94	*.32*	*.143*
*Social Acceptance (Bullying)*	−4.39	*.37*	*.082*	−5.69	*.46*	*.009*	−1.29	*.10*	*.855*
*Financial Resources*	−1.77	*.16*	*.729*	−4.28	*.37*	*.056*	−2.50	*.21*	*.371*

*MD*, mean difference; ES: Effect Size Cohen's d; *p*: *p*-values referring to significant group differences from planned contrasts; for clarity: scores refer to *t*-values, lower scores reflecting lower HRQoL in the index group compared against the respective reference group

### Psychopathological distress

Groups significantly differed on all dimensions of psychopathological distress, including *Somatization* (F_(2;260)_ = 10.61, *p < .0001*), *Obsessive-Compulsive* behavior (F_(2;260)_ = 19.31, *p < .001*), *Interpersonal Sensitivity* (F_(2;260)_ = 28.46, *p < .0001*), *Depression* (F_(2;260)_ = 27.76, *p < .0001*), *Anxiety* (F_(2;259)_ = 15.19, *p < .0001*), *Hostility* (F_(2;258)_ = 22.81, *p < .0001*), *Phobic Anxiety* (F_(2;259)_ = 9.38, *p < .0001*), *Paranoid Ideation* (F_(2;259)_ = 20.06, *p < .0001*), and *Psychoticism* (F_(2;259)_ = 20.13, *p < .0001*), as well as the *Global Severity Index* (F_(2;259)_ = 28.36, *p < .0001*). Group differences are illustrated in *Figure* 2 and descriptive statistics including pairwise contrasts and the respective effect size estimates are provided in Table 4. All effects remained after adjusting for sex and age in mixed-linear regression analysis. Sex only had a significant effect on *Interpersonal Sensitivity* and *Depression* (both greater in females). No effects of age on psychopathological distress were found.

**Table 4:** Group Contrast on Psychopathological Distress; Sidak corrected contrasts from one way analysis of variance

Dimension of Psychopathological Distress	no BPD vs. subthreshold BPD			no BPD vs. full-syndrome BPD			subthreshold BPD vs. full-syndrome BPD		
	MD	*ES*	*p*	MD	*ES*	*p*	MD	*ES*	*p*
*Somatization*	0.45	*-.55*	*.003*	0.57	-.69	.000	0.12	*-.15*	*.669*
*Obsessive-Compulsive*	0.55	*-.68*	*< .0001*	0.73	*-.94*	*< .0001*	0.18	*-.23*	*.313*
*Interpersonal Sensitivity*	0.74	*-.83*	*< .0001*	1.02	*−1.11*	*< .0001*	0.27	*-.32*	*.103*
*Depression*	0.77	*-.81*	*< .0001*	0.98	*−1.12*	*< .0001*	0.22	*-.26*	*.248*
*Anxiety*	0.50	*-.57*	*.002*	0.73	*-.84*	*< .0001*	0.24	*-.27*	*.179*
*Hostility*	0.60	*-.71*	*< .0001*	0.92	*−1.03*	*< .0001*	0.32	*-.35*	*.040*
*Phobic Anxiety*	0.38	*-.44*	*.027*	0.60	*-.66*	*< .0001*	0.21	*-.23*	*.282*
*Paranoid Ideation*	0.68	*-.81*	*< .0001*	0.84	*-.92*	*< .0001*	0.16	*-.18*	*.534*
*Psychoticism*	0.54	*-.69*	*< .0001*	0.77	*-.96*	*< .0001*	0.23	*-.28*	*.149*
*Global Severity Index*	0.58	*-.81*	*< .0001*	0.79	*−1.13*	*< .0001*	0.21	*-.32*	*.107*

*MD*, mean difference; ES: Effect Size Cohen's d; *p*: *p*-values referring to significant group differences from planned contrasts; for clarity: scores refer to scale scores that range from 0 (minimum) to 4 (maximum), greater scores reflecting greater psychopathological distress in the index group compared against the respective reference group

### Dimensional BPD, health related quality of life and psychopathological distress

Zero-order correlations (Table 5) showed, that the number of BPD criteria fulfilled was significantly and inversely related to all HRQoL dimensions except for *Social Support and Peers*. All domains of psychopathological distress were positively correlated with the number of BPD criteria. The frequency of self-injury was inversely related to the HRQoL dimensions of *Psychological Well-Being*, *Moods and Emotions*, *Self-Perception* and *Parent Relations and Home Life*. Further, frequency of self-injury was positively correlated with *Obsessive-Compulsive* symptoms, *Interpersonal Sensitivity*, *Depression*, *Anxiety*, *Paranoid Ideation*, *Psychoticism* and the *Global Severity Index* of psychopathological distress. The frequency of suicide attempts showed negative correlations with HRQoL in the dimensions of *Moods and Emotions*, *Social Acceptance (Bullying)*, and *Financial Resources*. Psychopathological distress and suicide attempts were not related. The number of BPD criteria met was positively associated with the frequency of self-injury (r_(235)_ = .156, *p = .017*) but not the number of suicide attempts (r_(130)_ = .061, *p = .492*). The frequency of self-injury and suicide attempts were not correlated (r_(126)_ = .111, *p = .213*).

**Table 5:** Clinical Concomitants of Health Related Quality of Life and Psychopathological Distress in Adolescents Engaging in Self-Injury

	BPD criteria	Self-injury	Suicide Attempts
Health Related Quality of Life			
*Physical-Wellbeing*	-.315***	-.108	-.143
*Psychological Well-Being*	-.364***	-.161**	-.115
*Moods and Emotions*	-.408***	-.153**	-.231**
*Self-Perception*	-.365***	-.219***	-.121
*Autonomy*	-.197**	-.064	-.015
*Parent Relations and Home Life*	-.292***	-.136*	-.115
*Social Support and Peers*	-.093	-.092	.025
*School Environment*	-.277***	-.111	-.090
*Social Acceptance (Bullying)*	-.212**	-.096	-.203*
*Financial Resources*	-.165**	.078	-.181*
Psychopathology Distress			
*Somatization*	.293***	.108	.074
*Obsessive-Compulsive*	.376***	.132*	.059
*Interpersonal Sensitivity*	.448***	.222***	.092
*Depression*	.438***	.204**	.110
*Anxiety*	.377***	.163*	.025
*Hostility*	.452***	.119	-.032
*Phobic Anxiety*	.302***	.111	.053
*Paranoid Ideation*	.359***	.150*	.148
*Psychoticism*	.401***	.176**	.153
*Global Severity Index*	.458***	.202**	.099

*BPD criteria*, number of BPD criteria fulfilled (0–9); Self-Injury: acts of self-injury within the past 12 months; Suicide Attempts: number of lifetime suicide attempts; analyses on BPD criteria based on *n* = 255 to 263; analyses on frequency of self-injury based on *n* = 215 to 235; analyses on suicide attempts based on *n* = 118 to 131

## Discussion

The present study aimed to investigate differences in sociodemographic and clinical characteristics of adolescents presenting with risk-taking and/or self-harming behavior without BPD compared to those with subthreshold and full-syndrome BPD. Treatment seeking adolescents who fulfilled diagnostic criteria for subthreshold or full-syndrome BPD were typically older. In line with the finding, that groups did not differ with respect to the time since first presentation in the professional mental health care system, results indicate that adolescents with full-syndrome BPD seek treatment about one year later compared to adolescents with no BPD. This finding highlights, that the age of 15 might characterize a critical window in the development and presentation of BPD symptoms. Adolescents with full-syndrome BPD were less likely to live with their biological mother – indicating differences in the familial background of those with full-syndrome BPD. None of the other sociodemographic variables, including educational status, studied within the present analyses showed differences between groups.

With respect to the clinical characteristics of the included patients, adolescents with subthreshold and full-syndrome BPD more frequently showed comorbid psychopathology. Previously, one study compared psychiatric comorbidity in adults with and without BPD and a history of NSSI. Findings showed that BPD is associated with greater diagnostic comorbidity, in line with the present findings [41]. The study found adults with BPD to be more likely to fulfill diagnostic criteria for anxiety disorders, but not mood, substance or psychotic disorders. Our findings highlight that mood disorders (F30-39) are most frequent in both, adolescents with subthreshold and full-syndrome BPD (~40%). Only psychoactive substance use (F10-19) and neurotic, stress-related and somatoform disorders (F40-48) were more frequent in adolescents with full-syndrome BPD. Findings of increased Axis I comorbidity in adolescent BPD are in line with previous studies in this age group [11], and findings in adults [42–44], particularly highlighting the importance of co-occurring mood disorders. Similar, findings on Axis II comorbidity are in line with previous studies in adolescents [11] and adults with BPD [45, 46], reporting that the most common comorbid Axis II disorders in BPD are dependent and avoidant personality disorder.

Frequency of NSSI significantly differed between groups, indicating that adolescents with full-syndrome BPD report greatest NSSI frequency. Findings on greater frequency of self-injury are in line with previous studies in college-based samples that showed higher rates of NSSI in undergraduate students with BPD compared to those without BPD [47]. Similar, in adults BPD is associated with more frequent NSSI [41]. In line with previous studies we found no difference in the age of NSSI onset [41]. The relative percentage of adolescents reporting at least one lifetime suicide attempt differed between groups, with ~70% of adolescents with full-syndrome BPD reporting previous suicide attempts. Important to note, that the number of total lifetime suicide attempts did not differ between groups.

Analyses of self-reports on HRQoL indicated greater burden in adolescents with subthreshold and full-syndrome BPD compared to adolescents presenting at a specialized outpatient clinic for risk-taking and self-harm behavior with no BPD. While all groups showed HRQoL below the normative mean of a representative and comparable sample of adolescents, those with BPD pathology (subthreshold and full-syndrome) showed decreased HRQoL compared to adolescents with no BPD on almost every domain of assessment with some exceptions when comparing adolescents with subthreshold BPD and no BPD. Most importantly, contradicting our hypothesis, adolescents with subthreshold and full-syndrome BPD did not differ on any domain of HRQoL, with the exception of *Self-Perception*, indicating that subthreshold BPD in adolescents is already associated with profound decreases in HRQoL.

Reduced HRQoL has previously been shown to improve in young BPD patients receiving DBT treatment [48], in adults with BPD receiving interpersonal psychotherapy [49], and in a pilot study in adults with BPD receiving Narrative Exposure Therapy (NET) [50]. Similar, there is very preliminary evidence that group schematherapy can improve HRQoL in adolescents with personality disorders [51]. Future studies, addressing the longitudinal course of HRQoL in adolescent subthreshold and full-syndrome BPD are necessary to investigate developmental domains underlying HRQoL and its mechanisms. Previous studies suggest, that longitudinal mood variability, assessed through real-time monitoring, is related to HRQoL in patients with BPD [52].

Analyses on dimensions of psychopathological distress revealed quite similar findings. Again, treatment seeking adolescents with risk-taking and self-harm behavior showed greater psychopathological distress compared to a representative sample of adolescents, independent of the presence of BPD symptoms. Although adolescents with subthreshold and full-syndrome BPD showed significantly increased psychopathological distress compared to adolescents with no BPD on all domains, contradicting our hypothesis, adolescents with subthreshold and full-syndrome BPD did not differ on measures of psychopathological distress, except for *Hostility*.

These findings highlight that beyond the DSM-5 diagnostic cut-off (i.e., fulfilling at least 5 of 9 criteria) subthreshold BPD (i.e., fulfilling at least 3 of 9 criteria) in adolescents is already associated with severe impairments in HRQoL and psychopathological distress. While including subthreshold BPD in both clinical and neurobiological studies among youth is a matter of ongoing debate [53], our data support the validity of subthreshold BPD among adolescents and highlight its clinical relevance. Together with our finding that adolescents with full-syndrome BPD seek medical treatment later than those with no BPD, these findings raise awareness on the importance and diagnostic validity of BPD traits even below the established clinical cut-off.

While the present data provide support for the clinical utility of lower clinical cut-offs in diagnosis adolescent BPD, they also lend support to a dimensional BPD construct in adolescents [18, 19]. In the present representative sample of treatment seeking adolescents, HRQoL and psychopathological distress were correlated with the total number of BPD criteria endorsed. Further, results illustrate that self-injurious behavior and suicide attempts are correlated with HRQoL and psychopathological distress. Somewhat surprising is the findings that suicide attempts are correlated with HRQoL but not self-reports of psychopathological distress. Although we can only speculate on the lack of association, in particular between self-reports of depression and suicide attempts, not the perceived severity of affective states but their actual impact in everyday life – as expressed by the HRQoL domain of *Moods and Emotions* – seems to be associated with the frequency of suicide attempts.

The study faces several limitations that need to be addressed. Our analyses are based on a consecutive treatment seeking sample of adolescents engaging in risk-taking behavior and self-harm. Thus, findings might not generalize to adolescents in general. Help-seeking is considerable low in adolescents engaging in self-injury and risk-taking [54, 55] and particularly low among males. While our outpatient clinic implements specific measures to increase help-seeking (i.e., open clinic) in order to lower the threshold for clinical presentation, adolescents presenting themselves at the clinic might represent a specific group. On the other hand, the large consecutive sample represents a major strength of the present study, reflecting a representative clinical situation. Our analyses highlight important differences in the perceived burden of adolescents with risk-taking behavior and/or self-injury. However, in addition to self-reports of HRQoL and psychopathological distress, the integration of additional ratings by parents and/or teachers presents an interesting avenue to future research. Finally, future studies assessing the validity of clinical cut-offs in the diagnostic assessment of BPD in adolescents, would do well to implement multi-method assessments of BPD.

## Conclusions

Both full-syndrome and subthreshold BPD in adolescents are associated with severe impairments in HRQoL and psychopathological distress. Based on these findings, the diagnostic standard that five of nine criteria fulfilled constitute a BPD diagnosis needs to be questioned in adolescent patients. Our findings support the inclusion of adolescents with a lower cut-off into research on adolescent BPD and highlight the necessity of early intervention in adolescent BPD.

## Abbreviations

BPD: Borderline Personality Disorder
DSM: Diagnostic and Statistical Manual for Mental Disorders
HRQoL: Health Rated Quality of Life
ICD: International Classification of Disease
M.I.N.I-KID: *Mini-International Neuropsychiatric Interview for Children and Adolescents*
MD: Mean Difference
NSSI: non-suicidal self-injury
SCID-II: *Structured Clinical Interview for DSM-IV-Axis II*
SCL-90-R: Symptom Checklist-90-R
SITBI-G: *German version of the Self-Injurious Thoughts and Behavior Interview*

## References

[1] Coid J, Yang M, Tyrer P, Roberts A, Ullrich S (2006). Prevalence and correlates of personality disorder in Great Britain. Br J Psychiatry J Ment Sci.

[2] Trull TJ, Jahng S, Tomko RL, Wood PK, Sher KJ (2010). Revised NESARC personality disorder diagnoses: gender, prevalence, and comorbidity with substance dependence disorders. J Personal Disord.

[3] American Psychiatric Association. Diagnostic and statistical manual of mental disorders (5th ed.). Arlington, VA: American Psychiatric Publishing; 2013.

[4] Leichsenring F, Leibing E, Kruse J, New AS, Leweke F (2011). Borderline personality disorder. Lancet.

[5] Chanen AM, McCutcheon LK (2008). Personality disorder in adolescence: The diagnosis that dare not speak its name. Personal Ment Health.

[6] Kaess M, Brunner R, Chanen A. Borderline Personality Disorder in Adolescence. Pediatrics. 2014;134(4):peds.2013-3677. 10.1542/peds.2013-3677 25246626

[7] Chanen AM (2015). Borderline Personality Disorder in Young People: Are We There Yet?. J Clin Psychol.

[8] Chanen AM, Jovev M, McCutcheon LK, Jackson HJ, McGorry PD (2008). Borderline Personality Disorder in Young People and the Prospects for Prevention and Early Intervention. Curr Psychiatr Rev.

[9] Miller AL, Muehlenkamp JJ, Jacobson CM (2008). Fact or fiction: diagnosing borderline personality disorder in adolescents. Clin Psychol Rev.

[10] Chanen AM, Jackson HJ, McGorry PD, Allot KA, Clarkson V, Yuen HP (2004). Two-year stability of personality disorder in older adolescent outpatients. J Personal Disord.

[11] Kaess M, von Ceumern-Lindenstjerna I-A, Parzer P, Chanen A, Mundt C, Resch F (2013). Axis I and II comorbidity and psychosocial functioning in female adolescents with borderline personality disorder. Psychopathology.

[12] Chanen AM, McCutcheon L (2013). Prevention and early intervention for borderline personality disorder: current status and recent evidence. Br J Psychiatry Suppl.

[13] Tyrer P, Crawford M, Mulder R (2011). ICD-11 Working Group for the Revision of Classification of Personality Disorders. Reclassifying personality disorders. Lancet.

[14] Lawrence KA, Allen JS, Chanen A (2011). A study of maladaptive schemas and borderline personality disorder in young people. Cogn Ther Res.

[15] Nakar O, Brunner R, Schilling O, Chanen A, Fischer G, Parzer P (2016). Developmental trajectories of self-injurious behavior, suicidal behavior and substance misuse and their association with adolescent borderline personality pathology. J Affect Disord.

[16] Muehlenkamp JJ, Claes L, Havertape L, Plener PL (2012). International prevalence of adolescent non-suicidal self-injury and deliberate self-harm. Child Adolesc Psychiatr Ment Health.

[17] Swannell SV, Martin GE, Page A, Hasking P, St John NJ (2014). Prevalence of nonsuicidal self-injury in nonclinical samples: systematic review, meta-analysis and meta-regression. Suicide Life Threat Behav.

[18] Michonski JD, Sharp C, Steinberg L, Zanarini MC (2013). An item response theory analysis of the DSM-IV borderline personality disorder criteria in a population-based sample of 11- to 12-year-old children. Pers Disord.

[19] Sharp C, Ha C, Michonski J, Venta A, Carbone C (2012). Borderline personality disorder in adolescents: evidence in support of the Childhood Interview for DSM-IV Borderline Personality Disorder in a sample of adolescent inpatients. Compr Psychiatry.

[20] Zimmerman M, Chelminski I, Young D, Dalrymple K, Martinez J (2011). Does DSM-IV already capture the dimensional nature of personality disorders?. J Clin Psychiatry.

[21] Revicki DA, Kleinman L, Cella D (2014). A history of health-related quality of life outcomes in psychiatry. Dialogues Clin Neurosci.

[22] Saarni SI, Suvisaari J, Sintonen H, Pirkola S, Koskinen S, Aromaa A (2007). Impact of psychiatric disorders on health-related quality of life: general population survey. Br J Psychiatry.

[23] Katschnig H (2006). Quality of life in mental disorders: challenges for research and clinical practice. World Psychiatry Off J World Psychiatr Assoc WPA.

[24] IsHak WW, Elbau I, Ismail A, Delaloye S, Ha K, Bolotaulo NI (2013). Quality of life in borderline personality disorder. Harv Rev Psychiatry.

[25] Narud K, Mykletun A, Dahl AA (2005). Quality of life in patients with personality disorders seen at an ordinary psychiatric outpatient clinic. BMC Psychiatry.

[26] Grambal A, Prasko J, Kamaradova D, Latalova K, Holubova M, Sedláčková Z (2016). Quality of life in borderline patients comorbid with anxiety spectrum disorders - a cross-sectional study. Patient Prefer Adherence.

[27] Scheiderer EM, Wood PK, Trull TJ (2015). The comorbidity of borderline personality disorder and posttraumatic stress disorder: revisiting the prevalence and associations in a general population sample. Borderline Personal Disord Emot Dysregulation.

[28] Perseius K-I, Andersson E, Asberg M, Samuelsson M (2006). Health-related quality of life in women patients with borderline personality disorder. Scand J Caring Sci.

[29] World Medical Association (2013). World medical association declaration of helsinki: Ethical principles for medical research involving human subjects. JAMA.

[30] Sheehan DV, Sheehan KH, Shytle RD, Janavs J, Bannon Y, Rogers JE (2010). Reliability and validity of the Mini International Neuropsychiatric Interview for Children and Adolescents (MINI-KID). J Clin Psychiatry.

[31] Sheehan DV, Lecrubier Y, Sheehan KH, Amorim P, Janavs J, Weiller E (1998). The Mini-International Neuropsychiatric Interview (M.I.N.I.): the development and validation of a structured diagnostic psychiatric interview for DSM-IV and ICD-10. J Clin Psychiatry.

[32] Fydrich T, Renneberg B, Schmitz B, Wittchen H (1997). Strukturiertes Klinisches Interview für DSM-IV Achse II: Persönlichkeitsstörungen (SKID-II).

[33] Salbach-Andrae H, Bürger A, Klinkowski N, Lenz K, Pfeiffer E, Fydrich T (2008). Diagnostic of personality disorders in adolescence according to SCID-II. Z Kinder Jugendpsychiatr Psychother.

[34] Nock MK, Holmberg EB, Photos VI, Michel BD (2007). Self-Injurious Thoughts and Behaviors Interview: development, reliability, and validity in an adolescent sample. Psychol Assess.

[35] Fischer G, Ameis N, Parzer P, Plener PL, Groschwitz R, Vonderlin E (2014). The German version of the self-injurious thoughts and behaviors interview (SITBI-G): a tool to assess non-suicidal self-injury and suicidal behavior disorder. BMC Psychiatry.

[36] Rathus JH, Wagner D, Miller AL. Psychometric Evaluation of the Life Problems Inventory, a Measure of Borderline Personality Features in Adolescents. J Psychol Psychother. 2015;5(4):1–9. doi:10.4172/2161-0487.1000198 .

[37] Ravens-Sieberer U, Gosch A, Rajmil L, Erhart M, Bruil J, Duer W (2005). KIDSCREEN-52 quality-of-life measure for children and adolescents. Expert Rev Pharmacoecon Outcomes Res.

[38] Schmitz N, Hartkamp N, Kiuse J, Franke GH, Reister G, Tress W (2000). The Symptom Check-List-90-R (SCL-90-R): a German validation study. Qual Life Res Int J Qual Life Asp Treat Care Rehab.

[39] Derogatis L. Manual for the Symptom Checklist 90 Revised (SCL-90R). Baltimore; 1986

[40] Derogatis LR, Unger R. Symptom Checklist-90-Revised. In: The Corsini Encyclopedia of Psychology. Hoboken: John Wiley & Sons, Inc.; 2010. http://onlinelibrary.wiley.com/doi/10.1002/9780470479216.corpsy0970/abstract. Accessed 30 Aug 2016.

[41] Turner BJ, Dixon-Gordon KL, Austin SB, Rodriguez MA, Zachary Rosenthal M, Chapman AL (2015). Non-suicidal self-injury with and without borderline personality disorder: Differences in self-injury and diagnostic comorbidity. Psychiatry Res.

[42] Grant BF, Chou SP, Goldstein RB, Huang B, Stinson FS, Saha TD (2008). Prevalence, correlates, disability, and comorbidity of DSM-IV borderline personality disorder: results from the Wave 2 National Epidemiologic Survey on Alcohol and Related Conditions. J Clin Psychiatry.

[43] Sar V, Akyuz G, Kugu N, Ozturk E, Ertem-Vehid H (2006). Axis I dissociative disorder comorbidity in borderline personality disorder and reports of childhood trauma. J Clin Psychiatry.

[44] Zanarini MC, Frankenburg FR, Dubo ED, Sickel AE, Trikha A, Levin A (1998). Axis I comorbidity of borderline personality disorder. Am J Psychiatry.

[45] Zanarini MC, Frankenburg FR, Vujanovic AA, Hennen J, Reich DB, Silk KR (2004). Axis II comorbidity of borderline personality disorder: description of 6-year course and prediction to time-to-remission. Acta Psychiatr Scand.

[46] Lenzenweger MF, Lane MC, Loranger AW, Kessler RC (2007). DSM-IV personality disorders in the National Comorbidity Survey Replication. Biol Psychiatry.

[47] Bracken-Minor KL, McDevitt-Murphy ME (2014). Differences in features of non-suicidal self-injury according to borderline personality disorder screening status. Arch Suicide Res Off J Int Acad Suicide Res.

[48] Swales M, Hibbs R A. B, Bryning L, Hastings RP. Health related quality of life for young people receiving dialectical behaviour therapy (DBT): a routine outcome-monitoring pilot. SpringerPlus. 2016;5:1137. 10.1186/s40064-016-2826-9 PMC4954798 27504235

[49] Bozzatello P, Bellino S (2016). Combined therapy with interpersonal psychotherapy adapted for borderline personality disorder: A two-years follow-up. Psychiatry Res.

[50] Steuwe C, Rullkötter N, Ertl V, Berg M, Neuner F, Beblo T (2016). Effectiveness and feasibility of Narrative Exposure Therapy (NET) in patients with borderline personality disorder and posttraumatic stress disorder - a pilot study. BMC Psychiatry.

[51] Roelofs J, Muris P, van Wesemael D, Broers NJ, Shaw I, Farrell J (2016). Group-Schematherapy for Adolescents: Results from a Naturalistic Multiple Case Study. J Child Fam Stud.

[52] Tsanas A, Saunders KEA, Bilderbeck AC, Palmius N, Osipov M, Clifford GD (2016). Daily longitudinal self-monitoring of mood variability in bipolar disorder and borderline personality disorder. J Affect Disord.

[53] Fonagy P, Speranza M, Luyten P, Kaess M, Hessels C, Bohus M (2015). ESCAP Expert Article: borderline personality disorder in adolescence: an expert research review with implications for clinical practice. Eur Child Adolesc Psychiatry.

[54] Doyle L, Treacy MP, Sheridan A (2015). Self-harm in young people: Prevalence, associated factors, and help-seeking in school-going adolescents. Int J Ment Health Nurs.

[55] Pumpa M, Martin G (2015). The impact of attitudes as a mediator between sense of autonomy and help-seeking intentions for self-injury. Child Adolesc Psychiatr Ment Health.

[56] The KIDSCREEN Group Europe. The KIDSCREEN questionnaires – Quality of life questionnaires for children and adolescents. Lengerich: Pabst-Science-Publishers; 2006.

[57] Essau C, Karpinski N, Petermann F, Conradt J. Häufigkeit und Komorbidität psychischer Störungen bei Jugendlichen: Ergebnisse der Bremer Jugendstudie. Z Für Klin Psychol Psychiatr Psychother. 1998;46:105–24.

[58] Franke G (2000). Die Symptom-Checkliste von Derogatis (SCL-90-R) - Deutsche Version - Manual.

